# Birth experience and early postpartum outcomes: A cross-sectional study of mother–infant bonding, breastfeeding self-efficacy, and depressive symptoms

**DOI:** 10.18332/ejm/217879

**Published:** 2026-02-27

**Authors:** Milda Naginevičiūtė, Eglė Bartusevičienė, Aurelija Blaževičienė

**Affiliations:** 1 Department of Nursing, Faculty of Nursing, Lithuanian University of Health Sciences, Kaunas, Lithuania; 2 Department of Obstetrics and Gynaecology, Faculty of Medicine, Lithuanian University of Health Sciences, Kaunas, Lithuania

**Keywords:** birth experience, breastfeeding self-efficacy, mother–infant bonding, postpartum depression, low-risk childbirth

## Abstract

**INTRODUCTION:**

Childbirth is a transformative experience with lasting psychological and emotional effects. Evidence shows that birth experience influences maternal outcomes such as breastfeeding self-efficacy, mother–infant bonding, and postpartum depression, yet these associations in low-risk births remain insufficiently explored. This study examined how birth satisfaction relates to early postpartum maternal well-being.

**METHODS:**

A cross-sectional study was conducted in Lithuania among 218 women who experienced low-risk deliveries. Data collection occurred between September 2022 and July 2024. The present analysis is based on data collected at 6–8 weeks postpartum. Standardized instruments were used to assess birth satisfaction (BSS-R), breastfeeding self-efficacy (BSES-SF), mother–infant bonding (MIBS), and postpartum depressive symptoms (EPDS). Mann–Whitney U tests and linear regression models were applied, with p<0.05 considered statistically significant.

**RESULTS:**

A positive birth experience was associated with stronger mother–infant bonding (less resentment, disappointment, and aggression; all p≤0.003) and higher protective emotions (p=0.025). These women also reported higher breastfeeding self-efficacy, including confidence in milk intake, latch, and managing difficulties (all p≤0.012). Negative experiences were linked to higher postpartum depressive symptoms (EPDS p=0.014), particularly anxiety and reduced enjoyment (both p≤0.008). Birth satisfaction predicted breastfeeding self-efficacy (R^2^=0.34; β=0.34, p<0.001, 95% CI: 0.63–1.39) and bonding (R^2^=0.30; β= -0.30, p<0.001, 95% CI: -0.16 – -0.07) but only weakly predicted postpartum depression (R^2^=0.09).

**CONCLUSIONS:**

A positive birth experience is associated with stronger mother–infant bonding and higher breastfeeding self-efficacy. Although linked to fewer depressive symptoms, its predictive value for postpartum depression was limited, indicating the importance of additional psychosocial determinants.

**ABBREVIATIONS:**

BSS-R: Birth Satisfaction Scale-Revised, BSES-SF: Breastfeeding Self-Efficacy Scale-Short Form, MIBS: Mother-Infant Bonding Scale, EPDS: Edinburgh Postnatal Depression Scale

## INTRODUCTION

Childbirth is a significant event in a woman’s life, with long-lasting psychological and physical effects^1^. In addition to physical changes, birth is a complex experience with significant emotional and psychological components that impact postnatal recovery, mother–infant bonding, mother self-confidence, and mental health^1,2^. Despite its importance, the psychological impact of childbirth remains underexplored compared to its medical aspects.

The World Health Organization (WHO) emphasizes the importance of a positive birth experience as a core element of maternity care^3^. Higher maternal satisfaction, enhanced mother–infant bonding, and more effective breastfeeding are closely associated with a positive birth experience^4^. Conversely, negative birth experiences have been linked to increased stress, the likelihood of traumatic birth, and postpartum depression – all of which may undermine maternal–infant bonding and overall maternal well-being^5-7^.

While previous research has primarily focused on physiological and clinical aspects, such as pain management, the effectiveness of medical interventions, and the reduction of the risk of obstetric complications^8,9^, psychological aspects of childbirth often receive insufficient attention, even though they are critical. Emotional subjective experiences during childbirth significantly affect maternal–infant bonding and maternal mental health^10-13^.

Postnatal depression affects approximately 10–20% of mothers in the first year postpartum, affecting not only maternal well-being but also child behavioral, emotional, and cognitive development^2,13^. In Lithuania, the number of officially registered cases is significantly lower. In 2022, only 0.18% of births were officially recorded as postnatal depression cases, suggesting that many cases remain undiagnosed^14^. This discrepancy suggests potential underdiagnosis, highlighting the need for a better understanding of maternal psychological well-being in the postnatal period^15^.

The birth experience is shaped not only by clinical events but also by subjective factors such as a sense of control, emotional support, access to information, and the overall birth environment – all of which are crucial to determining outcomes^7,16^. Research suggests that mothers who feel empowered and actively involved in decision-making during childbirth are more likely to have a positive birth experience, even when medical interventions are required^7,16-18^.

Furthermore, early postnatal interaction between mother and infant is crucial for establishing maternal–infant bonding and initiating breastfeeding^4,19^. Skin-to-skin contact during breastfeeding induces the release of oxytocin and strengthens the mother–infant bonding^19,20^. Mothers who define their birth experiences as positive are not only more inclined to initiate breastfeeding but also to breastfeed for longer^21^. Maternal mental health conditions, such as anxiety and depression, however, may affect the success of breastfeeding^22^.

Despite substantial research on birth experiences, studies integrating their effects on breastfeeding self-efficacy, mother–infant bonding, and postnatal depression remain limited. Understanding how these factors interact can provide valuable insights into improving maternal care and postnatal outcomes.

This study aims to evaluate the associations between birth experience and maternal outcomes, focusing on breastfeeding self-efficacy, mother–infant bonding, and postpartum depression in low-risk deliveries.

## METHODS

### Study design and participants

This cross-sectional study is part of a larger research project evaluating various aspects of the postpartum period among women with low-risk childbirth^23^. The present analysis was conducted to evaluate the impact of childbirth experience on maternal outcomes. In this study, maternal outcomes are defined as key indicators of maternal well-being, including mother–infant bonding, postpartum depressive symptoms, and breastfeeding self-efficacy.

Data collection occurred between September 2022 and July 2024 at 6–8 weeks postpartum. The study included women aged ≥18 years who were fluent in Lithuanian and experienced low-risk deliveries. Low-risk delivery was defined in accordance with the national methodology for normal childbirth^24^, referring to spontaneous term labor without obstetric or medical complications and with a minimal likelihood of medical interventions. Women whose deliveries transitioned from low-risk to intermediate or high-risk during labor or delivery, were excluded.

The study was conducted in two Lithuanian hospitals – a university hospital and a regional hospital – both of which provide maternity care for women with low-risk deliveries. These hospitals were selected to ensure a diverse clinical population and to reflect the maternity care settings available in Lithuania. Both hospitals manage low-risk deliveries within the framework of standard perinatal care, allowing for the examination of maternal experiences in routine childbirth settings.

Eligible participants were identified through hospital records and invited to participate by the research team during their postpartum hospital stay. A total of 218 women provided complete data at 6–8 weeks postpartum and were included in the present analysis. As this analysis was conducted within a broader research project, the sample size was predetermined based on methodological considerations for the main study; therefore, a separate sample size calculation was not performed for the present cross-sectional analysis.

### Data collection

Data for the present study were collected at 6–8 weeks postpartum using an online questionnaire. Participants who agreed to take part in the study provided their contact information and received a link to the questionnaire by email. They were instructed to complete the questionnaires independently in a quiet environment to avoid external influence. All responses were submitted electronically, ensuring confidentiality and data protection.

To maintain data quality, incomplete questionnaires were excluded from the analysis. The questionnaires were designed to be user-friendly, and participants were encouraged to contact the research team with any questions or concerns.

### Measurement

The data were collected using a subset of the questionnaire from a comprehensive survey developed by the International Consortium for Health Outcomes Measurement (ICHOM)^23^. Five standardized questionnaires were employed. These instruments were selected based on their validity and relevance to key aspects of the study, including birth experience, postpartum mental health, breastfeeding confidence, and mother–infant bonding. Table 1 provides an overview of the questionnaires, including their scoring systems and specific roles within this study.

**Table 1:** Overview of questionnaires used in the study, Lithuania, September 2022–July 2024 (N=218)

*Questionnaire*	*Purpose*	*Items*	*Scoring* *range*
BSES-SF	Measures breastfeeding self-efficacy	14	14–70
MIBS	Assesses mother–infant bonding	8	0–24
EPDS	Screens for postpartum depression symptoms	10	0–30
BSS-R	Measures satisfaction with the childbirth experience	10	10–50

BSES-SF: Breastfeeding Self-Efficacy Scale–Short Form. MIBS: Mother–Infant Bonding Scale. EPDS: Edinburgh Postnatal Depression Scale. BSS-R: Birth Satisfaction Scale–Revised.

The Breastfeeding Self-Efficacy Scale–Short Form (BSES-SF) was utilized to measure women’s confidence in their ability to breastfeed successfully. The scale, developed initially and psychometrically assessed by Dennis^25^, consists of 14 items rated on a 5-point Likert scale, with total scores ranging from 14 to 70. Higher scores indicate greater confidence in breastfeeding self-efficacy^25^.

The Mother–Infant Bonding Scale (MIBS) was used to assess mothers’ emotional responses toward their infants during the postnatal period. Developed by Taylor et al.^26^, the MIBS is an 8-item self-report scale designed to screen for early bonding difficulties. Each item is rated on a 4-point Likert scale ranging from 0 (‘not at all’) to 3 (‘very much’), with total scores ranging from 0 to 24. Higher scores indicate greater emotional difficulties in the mother–infant bond^26^.

The Edinburgh Postnatal Depression Scale (EPDS), developed by Cox et al.^27^, was used to screen for symptoms of postnatal depression in women after childbirth. Total scores range from 0 to 30, with higher scores indicating a greater likelihood of postnatal depression^27^. The scale has been extensively validated across different populations, including Lithuania^28^.

The Birth Satisfaction Scale-Revised (BSS-R), a validated multi-dimensional measure developed by Hollins-Martin and Martin^29^, assessed women’s satisfaction with their childbirth experience. Responses are scored on a 5-point Likert scale ranging from 0 (‘strongly disagree’) to 4 (‘strongly agree’), with total scores ranging from 0 to 50. Higher scores indicate greater satisfaction with the childbirth experience^29^. The participants were divided into two groups based on their childbirth experience: those with a positive experience and those with a negative experience. The groups were classified according to the BSS-R scale: Negative experience (BSS-R<28 points) and Positive experience (BSS-R ≥28 points)^29^.

The standardized questionnaires (MIBS, BSES-SF, and BSS-R) were translated and culturally adapted from English into Lithuanian with permission from the original authors. The translation process followed the ISPOR Principles of Good Practice for the Translation and Cultural Adaptation of Patient-Reported Outcome Measures, including forward translation, back translation, expert review, and iterative refinement to ensure semantic and conceptual equivalence. The adapted measures were then tested in a sample of postpartum women to assess comprehensibility, refine phrasing for clarity, and confirm cultural relevance.

### Statistical analysis

The statistical analysis of the study data was performed using SPSS/W 29.0. Descriptive statistics were applied, with results presented as frequencies (n) and percentages (%). For quantitative variables, the mean and standard deviation (SD), minimum, maximum, and median were calculated. Comparative statistics were used to test statistical hypotheses. Main study variables included birth satisfaction (BSS-R), breastfeeding self-efficacy (BSES-SF), mother–infant bonding (MIBS), postpartum depressive symptoms (EPDS), and sociodemographic characteristics (maternal age, education level, marital status, and parity). As only simple linear regression models were applied, no covariates or potential confounders were included in the analyses. The Mann-Whitney U test was applied to compare the distributions of non-parametric variables between two independent samples. The chi-squared test was used to compare categorical sociodemographic variables between groups. The relationships between birth satisfaction (BSS-R) and each maternal outcome (MIBS, BSES-SF, and EPDS) were analyzed using simple linear regression models. No multivariable regression models were applied in the present analysis. Model fit was assessed by calculating the coefficient of determination (R^2^), variance inflation factor (VIF), Cook’s distance, and maximum DFBeta values. A p<0.05 was considered statistically significant.

## RESULTS

A total of 218 women participated in the study. The mean age of participants was 31.0 ± 4.6 years. Among them, 45% (n=98) reported a positive birth experience and 55% (n=120) reported a negative birth experience. Most participants had non-university education (59.6%), were married (81.2%), and slightly more than half were multiparous (53.2%). No statistically significant differences were found between women with positive and negative birth experiences across sociodemographic characteristics, including age, education level, marital status, and parity (all p>0.05). These results are summarized in Table 2.

**Table 2:** Sociodemographic characteristics of participants according to birth experience, Lithuania, 2022–2024 (N=218)

*Characteristics*	*Total* *n (%)*	*Birth experience*		*p*
		*Negative* *n (%)*	*Positive* *n (%)*	
**Total**, n	218	120	98	
**Maternal age** (years), mean ± SD	31.00 ± 4.6	31.41 ± 4.6	30.58 ± 4.6	0.128
**Education level**				
Primary/secondary/vocational	53 (24.3)	27 (22.5)	26 (26.5)	0.285
Higher non-university	130 (59.6)	77 (64.2)	53 (54.1)	
Higher university	35 (16.1)	16 (13.3)	19 (19.4)	
**Marital status**				
Married	177 (81.2)	95 (79.2)	82 (83.7)	0.698
Cohabitation	38 (17.4)	23 (19.2)	15 (15.3)	
Single/divorced	3 (1.4)	2 (1.6)	1 (1.0)	
**Parity**				
Nulliparous	102 (46.8)	57 (47.5)	45 (45.9)	0.614
Multiparous	116 (53.2)	63 (52.5)	53 (54.1)	

P-values for education level, marital status, and parity were calculated using the chi-squared test; maternal age was compared between groups using the Mann–Whitney U test.

Mothers with a positive birth experience reported significantly stronger maternal–infant bonding, as indicated by lower levels of resentment (p=0.003), disappointment (p<0.001), and aggressive feelings toward the infant (p=0.001), as well as higher protective emotions (p=0.025) compared to those with negative birth experiences. These results are summarized in Table 3.

**Table 3:** Mother–infant bonding (MIBS) scores according to birth experience, Lithuania, 2022–2024 (N=218)

*MIBS statements*	*Birth experience*		*p*
	*Negative* *(N=120)* *Mean ± SD*	*Positive* *(N=98)* *Mean ± SD*	
Loving	0.07 ± 0.3	0.06 ± 0.4	0.076
Resentful	0.25 ± 0.6	0.10 ± 0.5	0.003
Neutral or felt nothing	0.13 ± 0.3	0.09 ± 0.3	0.340
Joyful	0.45 ± 0.6	0.35 ± 0.6	0.133
Dislike	0.22 ± 0.7	0.19 ± 0.4	0.218
Protective	0.29 ± 0.6	0.13 ± 0.4	0.025
Disappointed	0.27 ± 0.4	0.06 ± 0.2	<0.001
Aggressive	0.10 ± 0.3	0.00 ± 0.00	0.001

MIBS: Mother–Infant Bonding Scale; score range 0–24, higher scores indicate greater bonding difficulties. Negative birth experience: BSS-R<28. Positive birth experience: BSS-R ≥28. Statistical comparisons performed using the Mann–Whitney U test.

**Figure 1:**
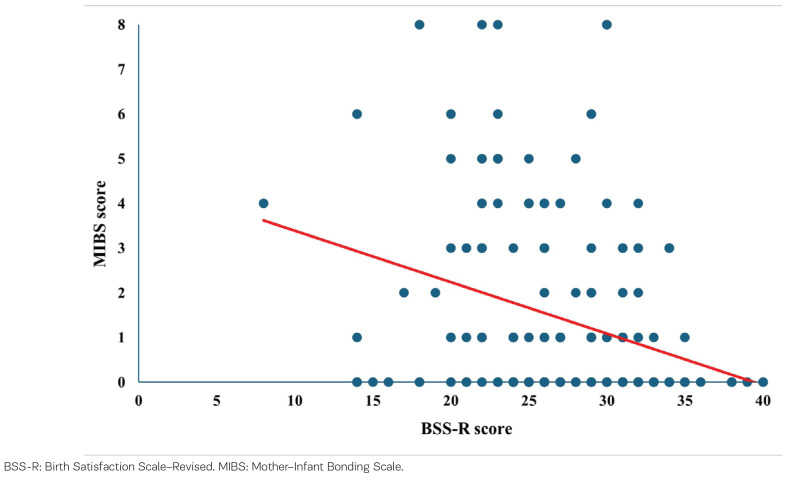
Linear regression between birth satisfaction and mother–infant bonding, Lithuania, 2022–2024 (N=218)

Linear regression analysis demonstrated that birth satisfaction (BSS-R) was a significant predictor of mother–infant bonding (MIBS) (R^2^=0.30; β= -0.30, p<0.001, 95% CI: -0.16 – -0.07). The relationship between birth satisfaction and mother–infant bonding is illustrated in Figure 1.

Breastfeeding self-efficacy was significantly higher among mothers who reported a positive birth experience. These mothers exhibited greater confidence in ensuring adequate milk intake (p<0.001), achieving proper latch (p=0.012), and managing breastfeeding challenges (p<0.001). Additionally, they reported higher confidence in handling the time demands of breastfeeding (p=0.003) and maintaining consistency in feeding (p=0.033). These results are summarized in Table 4.

**Table 4:** Breastfeeding self-efficacy (BSES-SF) scores according to birth experience, Lithuania, 2022–2024 (N=218)

*BSES-SF statements*	*Birth experience*		*p*
	*Negative* *(N=120)* *Mean ± SD*	*Positive* *(N=98)* *Mean ± SD*	
Determine that my baby is getting enough milk	3.63 ± 1.0	4.14 ± 1.1	<0.001
Successfully cope with breastfeeding like I have with other challenging tasks	3.47 ± 1.1	3.92 ± 1.2	<0.001
Breastfeed my baby without using formula as a supplement	3.83 ± 1.5	4.13 ± 1.4	0.146
Ensure that my baby is properly latched on for the whole feeding	3.50 ± 1.4	3.95 ± 1.3	0.012
Manage the breastfeeding situation to my satisfaction	3.49 ± 1.3	4.17 ± 1.3	<0.001
Manage to breastfeed even if my baby is crying	3.43 ± 1.3	3.91 ± 1.2	0.005
Keep wanting to breastfeed	3.69 ± 1.4	4.06 ± 1.3	0.037
Comfortably breastfeed with my family members present	3.57 ± 1.4	3.78 ± 1.3	0.357
Be satisfied with my breastfeeding experience	3.42 ± 1.4	4.10 ± 1.1	<0.001
Deal with the fact that breastfeeding can be time-consuming	3.74 ± 1.4	4.23 ± 1.2	0.003
Finish feeding my baby on one breast before switching to the other breast	3.67 ± 1.4	4.00 ± 1.2	0.140
Continue to breastfeed my baby for every feeding	3.93 ± 1.5	4.30 ± 1.3	0.033
Manage to keep up with my baby’s breastfeeding demands	3.81 ± 1.5	4.27 ± 1.3	0.012
Tell when my baby is finished breastfeeding	3.66 ± 1.3	4.17 ± 1.3	<0.001
Overall	50.83 ± 16.6	57.13 ±15.1	0.001

BSES-SF: Breastfeeding Self-Efficacy Scale–Short Form; score range 14–70, higher scores indicate greater breastfeeding self-efficacy. Negative birth experience: BSS-R<28. Positive birth experience: BSS-R ≥28. Statistical comparisons performed using the Mann–Whitney U test.

**Figure 2:**
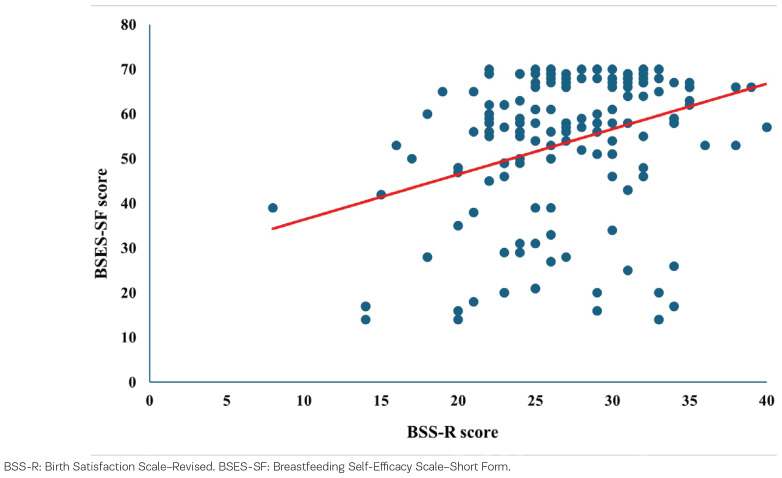
Linear regression between birth satisfaction and breastfeeding self-efficacy, Lithuania, 2022–2024 (N=218)

Birth satisfaction predicted breastfeeding self-efficacy. Linear regression analysis demonstrated that birth satisfaction (BSS-R) was a significant predictor of breastfeeding self-efficacy (BSES-SF) (R^2^=0.34; β=0.34, p<0.001, 95% CI: 0.63–1.39). The relationship between birth satisfaction and breastfeeding self-efficacy is illustrated in Figure 2.

Participants with negative birth experiences had significantly higher EPDS scores (7.01 ± 3.4) compared to those with positive experiences (5.74 ± 3.3; p=0.014). Symptoms of anxiety (p=0.008) and reduced enjoyment of daily activities (p<0.001) were particularly pronounced. Thoughts of self-harm were rare and did not show significant differences between the groups. These results are summarized in Table 5. Regression analysis revealed that birth experience (BSS-R) was not a significant predictor of postpartum depressive symptoms (EPDS) (R^2^=0.09).

**Table 5:** Postpartum depressive symptoms (EPDS) according to birth experience, Lithuania, 2022–2024 (N=218)

*EPDS statements*	*Birth experience*		*p*
	*Negative* *(N=120)* *Mean ± SD*	*Positive* *(N=98)* *Mean ± SD*	
I have been able to laugh and see the funny side of things	0.20 ± 0.4	0.10 ± 0.3	0.064
I have looked forward with enjoyment to things	0.30 ± 0.5	0.08 ± 0.3	<0.001
I have blamed myself unnecessarily when things went wrong	1.27 ± 0.9	1.19 ± 0.9	0.464
I have been anxious or worried for no good reason	1.83 ± 0.8	1.52 ± 0.9	0.008
I have felt scared or panicky for no good reason	1.27 ± 1.0	0.98 ± 0.9	0.066
Things have been getting to me	0.80 ± 0.7	0.70 ± 0.7	0.255
I have been so unhappy that I have had difficulty sleeping	0.22 ± 0.4	0.14 ± 0.4	0.203
I have felt sad or miserable	0.56 ± 0.6	0.49 ± 0.5	0.417
I have been so unhappy that I have been crying	0.06 ± 0.6	0.49 ± 0.5	0.490
The thought of harming myself has occurred to me	0.01 ± 0.1	0.04 ± 0.2	0.112
Overall	7.01 ± 3.4	5.74 ± 3.3	0.014

EPDS: Edinburgh Postnatal Depression Scale; score range 0–30, higher scores indicate more severe depressive symptoms. Negative birth experience: BSS-R <28. Positive birth experience: BSS-R ≥28. Statistical comparisons performed using the Mann–Whitney U test.

## DISCUSSION

The findings of this study underscore the association between birth experience and both mother–infant bonding and breastfeeding self-efficacy.

Our results showed that mothers who had a positive birth experience had a stronger emotional bond with their infant, with lower levels of negative emotions such as frustration and resentment. Protective emotions were also significantly higher, while feelings of aggression toward the baby were almost nonexistent in the positive birth experience group. These findings suggest that a positive birth experience may foster a stronger mother–infant emotional bond. It is consistent with findings from previous studies that maternal satisfaction with childbirth is associated with better emotional interactions with the infant and lower expressions of hostility^30,31^. Conversely, negative birth experiences can weaken the bond between mother and infant. Reviews have shown that women who rated their birth experience as negative were more likely to experience frustration, hostility, and emotional detachment^32^. In addition, it was observed that mothers who felt disempowered to make decisions or experienced a lack of control during childbirth were more likely to report a weaker bond with their infant^33^. This suggests that the objective parameters of childbirth and the mother’s subjective perception of the experience may significantly impact the early mother–infant bond.

Breastfeeding plays a crucial role in a mother’s confidence in her ability to care for her infant^31,34^. Our analysis revealed that a positive birth experience was a significant predictor of breastfeeding confidence, indicating that mothers who felt empowered and supported during childbirth were more confident in their ability to breastfeed successfully. This can be explained through the lens of successful experiences, which enhance a person’s confidence in their abilities^31,34^. The support received during childbirth, and the opportunity to be actively involved in decision-making, may strengthen the mother’s self-efficacy, which, in turn, reflects her confidence in her ability to breastfeed^31,34^. Our study showed that women with a positive birth experience demonstrated significantly higher confidence levels in breastfeeding across a range of dimensions. They were more confident in their ability to ensure adequate latch-on during breastfeeding, to effectively address feeding challenges, and to recognize their baby’s nutritional needs. These findings support previous research showing that a positive birth experience is associated with higher breastfeeding self-efficacy and a higher likelihood of continuing breastfeeding for longer^35^.

The experience of childbirth has long been considered one of the most important factors influencing a mother’s emotional state after childbirth^10,18,32^. The results of this study showed that women who had a negative birth experience were significantly more likely to experience increased symptoms of postnatal depression, particularly anxiety and reduced enjoyment of daily activities. These results are aligned with previous research suggesting that negative birth experiences may increase the risk of postnatal depression, particularly if women experience a lack of control, fear, or lack of emotional support during childbirth^32^. While these findings align with previous studies, some research suggests that the strongest risk factors for postpartum depression include psychosocial stressors, history of mental illness, and lack of social support^2,32,33^. The relatively low predictive value of birth satisfaction for postpartum depression in our study suggests that additional factors should be considered when assessing maternal emotional well-being in the postpartum period^33^. Research also shows that symptoms of postnatal depression tend to be most strongly felt in the first weeks after birth^33^. This may explain why, in the present study, in which depressive symptoms were assessed at 6–8 weeks, the influence of the birth experience was already less expressed.

It is also essential to consider that only women with low-risk pregnancies were included in this study. Low-risk births tend to be associated with a lower possibility of medical interventions, better postnatal physical health, and a greater sense of autonomy during childbirth, which may lead to a lower incidence of postnatal depression^3,9,10,32^. In contrast, women who have experienced complicated or traumatic births are more likely to have a stronger emotional impact on the birth experience and are at higher risk of developing postpartum depression symptoms^33^. This may explain why the impact of birth experience on depression scores was less pronounced in this study than in a previous study that included higher-risk births^36^.

### Strengths and limitations

This study provides comprehensive evidence on the impact of birth experience on mother–infant bonding, breastfeeding self-efficacy, and symptoms of postnatal depression. The use of validated instruments, such as the BSS-R, BSES-SF, MIBS, and EPDS, ensures the reliability of the results and facilitates comparisons with other international studies. Additionally, including only low-risk mothers reduces the influence of confounding factors, such as medical interventions or birth complications, allowing for a more precise assessment of the direct impact of birth experience on the analyzed aspects. The study is part of a broader research initiative and uses the International Consortium for Health Outcomes Measurement (ICHOM) methodology.

However, the study has some limitations. The study was conducted in only two Lithuanian hospitals, which limits the generalizability of the results to the broader national or international population. In addition, the study relied on self-reported data, which may have introduced information bias and potential misclassification. Furthermore, residual confounding cannot be excluded. Further research using larger and more diverse samples is needed to extend these findings.

## CONCLUSIONS

This study demonstrates that a positive birth experience is significantly associated with stronger mother–infant bonding and higher breastfeeding self-efficacy among women with low-risk deliveries. Women with positive birth experiences also reported fewer negative emotions toward their infant and greater confidence in breastfeeding. While negative birth experiences were linked to higher postpartum depressive symptoms, particularly anxiety and reduced enjoyment of daily activities, birth satisfaction had a weaker predictive value for postpartum depression than for mother–infant bonding and breastfeeding self-efficacy. Further research is needed to confirm these findings.

## Data Availability

The data supporting this research are available from the authors on reasonable request.
